# Oral Health Status of Ngäbe-Buglé Children in Panama: A Cross Sectional Study

**DOI:** 10.3390/children10020294

**Published:** 2023-02-02

**Authors:** Eliza R. C. Hagens, Sofia Maddalena Preatoni, Elena M. Bazzini, Daniel Akam, Konrad S. McKalip, Ben LaBrot, Maria Grazia Cagetti

**Affiliations:** 1 Floating Doctors, Bocas del Toro 0101, Panama; 2 Department of Biomedical, Surgical and Dental Sciences, University of Milan, Via Beldiletto 1, 20142 Milan, Italy; 3 Keck School of Medicine, University of Southern California, Los Angeles, CA 90089, USA

**Keywords:** child, dental caries, epidemiology, health status disparities, public health, indigenous oral health

## Abstract

Background: Poor oral health is often more prevalent in rural and resource-limited areas. Evaluating oral health status in these communities is the first step in ensuring adequate future health care for the population. The aim of this study was to assess the oral health status of children aged 6–12 years living in the indigenous Ngäbe-Buglé communities. Methods: A cross-sectional study was conducted in two rural indigenous communities of Ngäbe-Buglé on San Cristobal Island in Bocas del Toro, Panama. All children between 6 and 12 years of age and attending local schools were invited to participate, and those whose parents provided oral consent were enrolled. Dental examinations were performed by one trained dentist. To describe oral health, plaque index, DMFT/dmft (decayed, missing, and filled for permanent and primary teeth) index, and developmental defects of enamel index were recorded. Orthodontic characteristics were also evaluated, assessing the prevalence of different molar classes and the prevalence of open bite, lateral crossbite, and scissor bite. Results: A total of 106 children, representing 37.3% of the child population in the age range attending local schools, were included in this study. The mean plaque index of the entire population was 2.8 (SD 0.8). Caries lesions were more common in children living in San Cristobal (80.0%) compared to those living in Valle Escondido (78.3%), *p* = 0.827. The mean DMFT/dmft for the entire population was 3.3 (SD 2.9). Developmental defects of enamel were recorded in 49 children (46.2%). The majority of the population had a class I molar relationship (80.0%). Anterior open bite, lateral crossbite, and anterior crossbite were found in 10.4%, 4.7%, and 2.8% of the participants, respectively. Conclusions: The oral health of children living in Ngäbe-Buglé communities is generally poor. Oral health education programs for children and adults might play a crucial role in improving the oral health status of the Ngäbe-Buglé population. In addition, the implementation of preventative measures, such as water fluoridation as well as regular toothbrushing with fluoridated toothpaste and more accessible dental care, will be essential in improving future generations’ oral health.

## 1. Introduction

Good oral health is a fundamental aspect of overall wellbeing [1]. According to the WHO, approximately 2 billion people suffer from caries of the permanent teeth and 520 million children from caries of the primary teeth, with a higher prevalence of oral diseases in less developed areas [2]. This is likely related to a wide variety of factors, including lack of access to oral hygiene products or preventive dental care, infrastructural deficiencies such as no fluoridated drinking water, and lifestyle changes (i.e., increasing sugar consumption) and socioeconomic status. Geographic isolation and increased availability of sugary foods and beverages contribute to a growing burden of caries and other non-communicable diseases [3].

In underdeveloped areas, people often do not have access to dental care; in addition, adequate health facilities may be lacking. The relatively high cost of oral health treatment is a major barrier to accessing care. This further contributes to the deterioration of oral health, which in itself can lead to an increased risk of impoverishment [4,5]. 

In Panama, the Ministry of Health (MINSA) offers free basic dental care for those who are not covered by the social security fund (Caja de Seguro Social). However, access to these services is limited in rural areas, a problem exacerbated by high travel costs [6]. Isla San Cristobal is one of the closest islands to the urbanized area of Bocas Town. This facilitates access to health services compared to other communities in the region, most of which face a boat trip of over an hour to reach the nearest health care facility. 

The majority of Panama's population is mixed-race mestizo, but there are also several indigenous groups, the largest of which are the Ngäbe, closely affiliated with the Buglé group. Health disparities between the indigenous population and the rest of the population have been reported, including life expectancy and maternal mortality [7,8]. Data on oral health of the Ngäbe-Buglé population is lacking, and only one study has reported oral infections in pregnant and lactating women, showing a high prevalence of caries [9]. In 2008, a rather high mean decayed, missing, and filled teeth index (DMFT = 3.60) was collected among Panamanian children aged 12 years, demonstrating the burden of disease and the need for preventive and therapeutic interventions [10]. 

No large study has yet been conducted to evaluate oral health in Ngäbe-Buglé children. Epidemiological data can help to quantify the burden of oral diseases in this population. The non-governmental organization (NGO) Floating Doctors provides free health care to the Ngäbe-Buglé communities in this area and was therefore able to facilitate research on this population [11]. This epidemiological survey represents the first step in ensuring adequate oral health care, as it will provide a basis for future research, evidence for greater allocation of healthcare resources, and a metric with which the impact of interventions may be assessed. Therefore, the primary aim of this study is to assess the oral health status of children aged 6–12 years living in the Ngäbe-Buglé communities on San Cristobal Island, Bocas del Toro, Panama. The secondary aim is to assess the orthodontic characteristics in this population.

## 2. Materials and Methods

This is a cross-sectional study carried out on children aged between 6 and 12 years from Ngäbe-Buglé communities living in San Cristobal Island, Bocas del Toro, Panama.

Permission to conduct this survey was obtained from the school boards where the children attended school, and approval was obtained from the Ministry of Health in Panama (MINSA). This research was conducted in collaboration with the non-governmental organization (NGO) Floating Doctors, which provides free health care to the Ngäbe-Buglé communities [11]. As adults in these communities often cannot read or write, oral informed consent was obtained from the parents of all participants.

### 2.1. Study Population 

The Ngäbe-Buglé are one of several indigenous groups living in Panama, who together make up 12% of the Panamanian population [6]. The Ngäbe-Buglé communities are mostly located in the predominantly rural and mountainous western provinces of Chiriquí, Veraguas, and Bocas del Toro. All children who were between 6 and 12 years of age, living in the two Ngäbe-Buglé communities of Valle Escondido and San Cristobal on San Cristobal Island, Bocas del Toro, Panama, and attending local schools (total number of children *n* = 284) were invited to participate. Local communities drink rainwater, whose fluoride content, although hypothetically very low, is unknown.

The only additional inclusion criterium was that parents must have given oral informed consent. Children who did not offer sufficient compliance with the oral examination were excluded from the study. 

### 2.2. Study Procedures

Both communities in this study do not have direct access to regular oral health care. Floating Doctors visits the inhabitants of these communities once every three months to provide medical services. Dental treatment is only offered occasionally, when a dentist is available. The oral examinations were carried out on the same day as a medical clinic during June 2022, but in separate premises. 

Consenting parents were asked to bring their children to the school for the oral examination and data collection. The visits took place during school holidays, so no school days were missed. After registration of the participants, general demographic information was collected, including: community, age, sex, height (measured in meters), weight (measured in kilograms on an electric scale), systemic diseases, medication use, any previous dental visits, oral hygiene habits, and consumption of high-sugar foods and drinks. Height and weight were measured using a mechanical physician scale with integrated measuring rod (Rice Lake RL-MPS, Rice Lake, WI, USA). Appendix A shows the questionnaire in Spanish that each participant was asked to answer. Since all the children were enrolled in schools where the official language is Spanish, it can be assumed that they understood the proposed questions.

Following the collection of anamnestic data and the administration of the questionnaire, a calibrated dentist assessed oral health and orthodontic characteristics. All assessments were carried out according to the guidelines of the British Association of the Study of Community Dentistry (BASCD) [12]. All children were examined seated in a chair, using a dental mirror, a dental probe, and a headlamp. The data of each child were collected in an anonymized database. After the examination, each child participated in an oral health education lesson.

### 2.3. Assessment of Oral Health

Oral health status was assessed using plaque index, DMFT/dmft index, and DDE index. The plaque index as visible plaque was recorded as follows: absent, thin cervical film detectable with the probe, moderate accumulations visible to the naked eye, abundant accumulations, and bleeding on probing [13]. Data were collected for each sextant, and the tooth with the highest score was used as the score for the entire sextant. The plaque index was then calculated. The resulting scores was interpreted as follows: 0 = excellent hygiene; 0.1–0.9 = good hygiene; 1.0–1.9 = fair hygiene; and 2.0–3.0 = poor hygiene [13].

The teeth were then cleaned and dried with sterile gauze for subsequent inspection. The DMFT/dmft index as the sum of the number of D/d (decayed), M/m (missing) due to caries, and F/f (filled) teeth in the permanent/primary teeth was recorded [13]. For mixed dentition, DMFT and dmft were measured respectively and then added together [14]. 

Enamel defects on the buccal surfaces of anterior teeth and buccal and occlusal of posterior teeth were recorded according to the criteria described by the modified Dental Defects of the Enamel (DDE) index proposed by the FDI World Dental Federation, 1992 [15]. Enamel defects were classified as follows: no defect, demarcated opacities, diffuse opacities, and hypoplasia. The presence and severity of dental fluorosis was also investigated, using the Dean index [16].

### 2.4. Assessment of Orthodontic Characteristics

In subjects in whom the central permanent incisors and first molars were present, the following orthodontic characteristics were assessed: the different sagittal molar relationship, the presence of anterior and/or lateral open bite, the presence of lateral crossbite (unilateral or bilateral), and the presence of scissor bite (unilateral or bilateral).

First of all, the sagittal relationship of the first permanent molars, as described by the angle classification, was registered [17]. It was not recorded if the first permanent molars were missing. Second of all, the presence of anterior open bite was recorded if there was no vertical overlap of the incisors. A visible space between fully erupted canines, premolars, or molars with antagonists was registered as lateral open bite. Third of all, lateral crossbite was recorded if one or more buccal cusps of the mandibular canines, premolars, and/or molars occluded buccally to the buccal cusps of the maxillary antagonists in habitual occlusion. Finally, scissor bite was assessed if a total maxillary buccal (or mandibular lingual) crossbite was observed, with the mandibular dentition completely contained within the maxillary dentition in habitual occlusion [17].

### 2.5. Statistical Analysis

Continuous parametric data were reported as mean with respective standard deviations and continuous non-parametric data with medians, lower, and upper quartiles. Categorical data were reported as both number of observations and percentage. Body mass index (BMI) was calculating using child's height (in meters) and weight (in kilograms), using the formula BMI = kg/m^2^.

Plaque indices per patient were calculated as the sum of plaque indices for each sextant divided by 6. Statistical differences of plaque indexes between communities were compared using the Student’s *t*-test.

DMFT/dmft was calculated as the sum of decayed, missing, and filled teeth. For permanent, mixed, and primary dentition, DMFT and dmft were measured respectively and then summed. The average percentage of untreated carious lesions was calculated as D/DMFT or d/dmft, respectively. Statistical differences of DMFT/dmft indices between communities were compared using the Wilcoxon signed-rank test. 

The prevalence of developmental defects of enamel was calculated by dividing the number of children with enamel defects by the total number of children examined. The number of children with and without enamel defects were compared using the chi-square test. 

Differences in sagittal molar occlusion classes, incidence and severity of open bite, incidence of lateral crossbite, and incidence of scissor bite between communities were compared using the chi-square or Fisher’s exact test, if appropriate.

Missing data was handled with Complete Case Analysis. All *p*-values were based on a two-sided test, and *p*-values of <0.05 were considered statistically significant. All data were analyzed using IBM SPSS Statistics for Windows version 25 (IBM Corp., Armong, NY, USA) and R software (v 3.3.3).

## 3. Results

**Figure 1:**
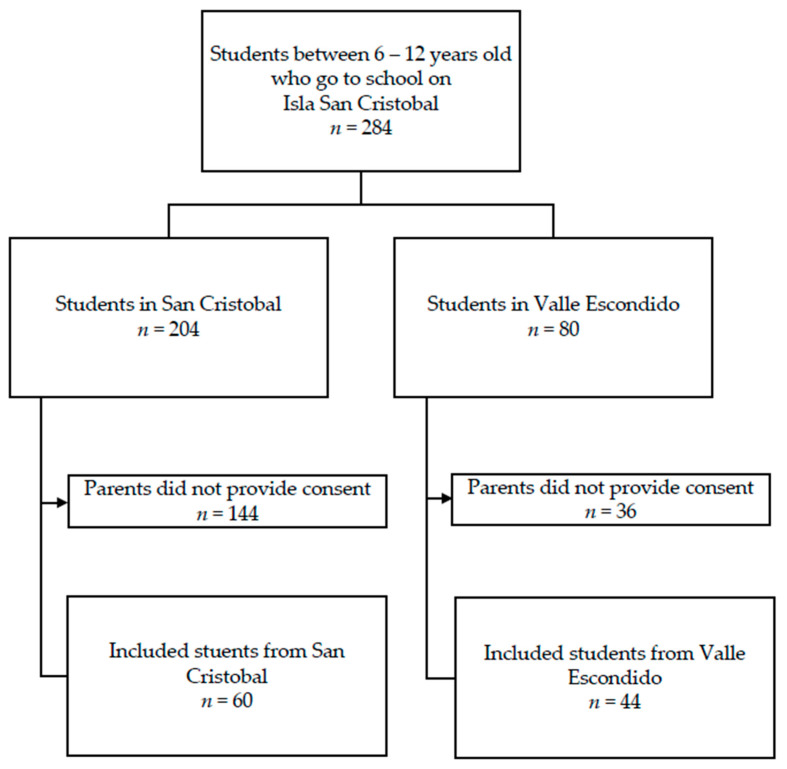
Flow chart of selection of included participants.

Of the 284 potential participants, 106 children whose parents provided informed consent underwent the oral examination (60 from San Cristobal and 46 from Valle Escondido, Figure 1). Characteristics of the included participants are shown in Table 1. Compared to children in San Cristobal, children in Valle Escondido had a statistically significant higher BMI (*p* = 0.018), and for a higher percentage of children, this was their first visit or examination by a dentist ever (*p* = 0.007). In Valle Escondido, a lower percentage of children drank hot chocolate on a daily basis (21.7%), compared to San Cristobal (51.7%) (*p* = 0.002). One participant was mute and could not answer the questions about oral hygiene habits and sugar intake. All children enrolled participated satisfactorily, so there were no missing data in the outcomes measured.

### 3.1. Oral Health Outcomes

The plaque index was similar in children from the San Cristobal and Valle Escondido communities (mean difference 0.08, *p* = 0.505). In both communities, more than half of the participants achieved a ‘fair hygiene’ plaque index (Table 2).

The overall prevalence of caries in the total sample was 79.2%, with a higher value in children from San Cristobal (80.0%) than in children from Valle Escondido (78.3%), although the difference was not statistically significant different (*p* = 0.827). The mean DMFT/dmft for the entire population was 3.3 ± 2.9. There was no statistically significant difference in DMFT/dmft between children from San Cristobal and those from Valle Escondido (3.1 ± 2.6 *versus* 3.6 ± 3.2, respectively; *p* = 0.520). The mean dmft in children from these communities was, respectively, 3.5 ± 2.7 and 0.8 ± 1.4 (*p* = 0.378). Overall, caries prevalence was higher in primary teeth than in permanent teeth (6.4% *versus* 34.0%, *p* = 0.005). Caries prevalence and DMFT/dmft scores are shown, combined and separately, in Table 3. 

**Figure 2:**
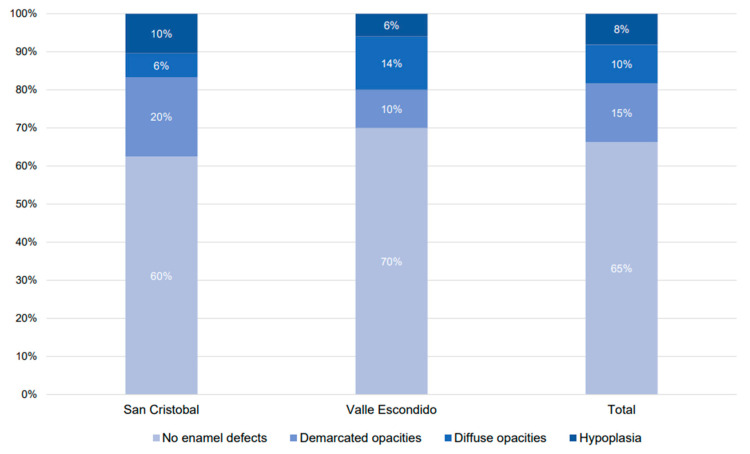
Percentage of teeth with and without developmental defects of enamel.

The total number of participants with developmental defects of enamel was 49 (46.2%). In San Cristobal, 29 (27.4%) children with enamel defects were found, compared to 20 (18.9%) detected in Valle Escondido (*p* = 0.619). Assessing the percentage of affected teeth, 15% presented demarcated opacities, 10% diffuse opacities, and 8% hypoplasia, while 65% showed no sign of defect. Differences between the communities are shown in Figure 2. There was no dental fluorosis seen in the study population.

### 3.2. Orthodontic Characteristics

In both Valle Escondido and San Cristobal, the majority of the children had a class I sagittal molar relationship. The percentage of participants with class I, II, and III sagittal molar relationships in Valle Escondido and San Cristobal were 81.8% *versus* 78.3%, 13.0% *versus* 6.7%, and 4.3% *versus* 15%, respectively, with no statistically significant difference (*p* = 0.122). Anterior open bite, lateral crossbite, and anterior crossbite in the two communities were also not statistically different (*p* = 0.521, *p* = 0.230, and *p* = 0.578, respectively). All orthodontic characteristics of the children are shown in Table 4.

## 4. Discussion

Few data have been published on the oral health status of the Ngäbe-Buglé population in Panama [8,18]. This population lives in remote and resource-poor communities with little or no support services, especially in the area of oral health. This study aimed to describe oral health status in a population of Ngäbe-Buglé children. Oral hygiene was generally poor in both communities included in this study, as evidenced by a mean plaque index of 2.8. One of the reasons for this low level of oral hygiene may stem from a lack of knowledge, in both adults and children, about proper oral hygiene practices. This situation calls for intervention aiming to improve the level of oral hygiene in this pediatric population in order to prevent diseases of the oral cavity, such as periodontal disease, in adulthood [19].

The prevalence of caries found in Ngäbe-Buglé children was high, with an overall average DMFT/dmft index of 3.3 and values above 10 in children with mixed dentition. No differences were found in the number of caries recorded in children in the two communities studied, demonstrating that caries are a serious health problem in the entire pediatric population. A previous study conducted in the province of Chiriquí Grande on 12-year-old Ngäbe-Buglé children showed a mean DMFT of 4.8 [18]. These data are not entirely comparable with the data collected in the present survey, since the present investigation refers to a sample of schoolchildren aged between 6 and 12 years. However, the average dmft values recorded show values not far from those recorded in the province of Chiriquí Grande. In both cases, the characteristics of the territory, an island in the present study and a mountainous territory in the previous study, may create logistical barriers that limit access to dental care. However, it should be noted that the study in Chiriquí Grande was conducted in 1998, when the infrastructure was less developed than today. To date, communities located in Chiriquí Grande have overall greater access to transport, services, and financial purchasing power than in the most remote Caribbean side, which includes San Cristobal Island. Furthermore, in the WHO database of Oral Health Country/Area Profile Project, the DMFT index reported in 12-year-old Panamanian children is 3.6 [8]. Again, comparisons cannot be made with the age-diverse population of this study. The sample in the WHO database does not refer to a rural community and included only boys; moreover, data was collected in 2008, ten years after the previous paper. What emerges is certainly a high caries prevalence that does not seem to have changed in over twenty years. There are several potential reasons why oral health in the study population was poor. Dental services, although free for the indigenous population, are not easily accessible because they are located in hospitals that may be hours away. This statement agrees with the data collected, which show that more than two-thirds of the sample examined had never been seen by a dentist. 

Oral health education is almost absent and is only provided sporadically by a few NGOs working in the area, such as Floating Doctors. It is therefore not surprising that more than two-thirds of the study population do not brush their teeth daily and sugar intake is very high, which could be a sign of a general lack of knowledge on oral health issues. Health education is extremely valuable, as shown by a study carried out on adolescents in Nepal, in which the study group that received oral hygiene lessons showed a significant improvement in plaque index compared to a control group that had not received any education [20]. The implementation of an education program could be an important resource for improving the oral health of the Ngäbe-Buglé population as well. In addition, although the daily diet of rural communities is mainly based on rice, beans, meat, and tropical fruits, the availability and thus consumption of junk food has increased in recent decades [21]. A study on the oral health status of indigenous communities in Ecuador showed a higher incidence of caries in those who had easy access to processed foods compared to those who did not [22]. In both communities surveyed, there is at least one grocery shop selling food and drinks high in processed sugars. It is therefore not surprising that almost all of the sample reported regularly consuming these types of foods and beverages.

Economic factors contribute to poor oral health. During the visit, some parents reported that they did not have the means to regularly buy toothpaste and toothbrush for their children; in fact, very often, the participants reported not owning a toothbrush. This situation may have been worsened by the recent COVID-19 pandemic, due to which toothbrushes and toothpaste have become more difficult to obtain. This may explain why more than one-third of the sample did not brush their teeth daily. 

With regard to developmental defects of enamel, demarcated opacities were the most frequently encountered defects, while hypoplasia was the least represented. No cases of dental fluorosis were recorded; fluoride intake in these communities is low, due to the usage of rainwater and the low use of fluoride toothpastes and other fluoridated preventive products. In addition, the observation conditions did not allow for air-drying of the tooth surfaces, and therefore questionable or very mild forms of fluorosis could have been missed. 

The prevalence of the different variables discussed so far could be partially distorted by the fact that many of the children enrolled had mixed dentition with varying numbers of deciduous and permanent teeth. Certainly, it would have been more correct from an epidemiological point of view to enroll a homogeneous sample in terms of age, but when assessing the oral condition of an ethnic minority, such as the one evaluated in this study, this limitation is difficult to overcome.

Although malocclusion cannot be considered a disease, but a developmental disorder, the poor relationship of the dental arches can negatively affect oral functions causing various problems such as occlusal trauma, temporomandibular disorder, reduced chewing performance, and impaired phonation that, if left untreated, persist throughout life [23]. The most frequent occlusal relationship recorded was class I. This figure is comparable to that reported in a recent systematic review that analyzed the prevalence of the different type of malocclusions globally, where a mean prevalence of 74.7% was found in mixed dentition. The prevalence of class II, on the other hand, was lower in the present sample than the global values, while that of class III occlusion was higher [24]. However, it must be remembered that the molar relationship can change from mixed dentition to permanent dentition [25]. The relationship between ethnicity and orthodontic features is well established, as the development of a particular facial bone pattern modifies orthodontic features. Somatic features of a prominent jaw, wide dental arches, and wide cheekbones are common in indigenous populations in Latin America [26]. These features promote normal dentition development, resulting in a harmonious occlusion. Orthodontic characteristics can also be influenced by childhood habits. Prolonged thumb sucking can cause stunted mandibular growth and a narrower, higher palate shape. This can result in a class II malocclusion and dental crowding due to a vertical rather than horizontal expansion of the palatine vault [27]. Although the questionnaire administered in the present survey did not include questions on non-nutritive sucking, field experience and interviews with inhabitants of Ngäbe-Buglé communities suggest that the thumb-sucking habit is not common in children, which may help to explain the low prevalence of class II occlusions found in this children population. 

This study has some limitations. Data on lifestyle habits were collected directly from the children, and therefore their reliability could be questioned, especially among younger participants. In addition, data on access to care and dental products in relation to the COVID-19 pandemic were not collected. The availability of dental care and products was even more limited, and this might have influenced the results. Furthermore, the findings of the present study may not be generalized to the entire Ngäbe-Buglé pediatric population, as the survey was conducted on communities that live relatively close to health services. This could lead to an overestimation of the oral health status of the entire Ngäbe-Buglé population. However, the data showed a reduced prevalence of children who had previously visited a dentist. It is therefore reasonable to assume that while more remote communities might be affected by factors such as reduced access to sugary foods, other factors such as lack of access to dental care might have an even greater impact than they do on communities close to the city, such as those included in this study. 

This is, to the best of authors’ knowledge, the only recent study that describes the current oral health status in the Ngäbe-Buglé population. This study shows that the oral health status of this population is of concern and that oral health promotion and prevention programs need to be implemented. Increased oral health education and other preventive measures such as fluoride intake can contribute to this goal.

## 5. Conclusions

The oral health of the study population was generally poor, a result that can probably be generalized to the entire population of Ngäbe-Buglé children living in Panama. Oral health education programs aimed at children and adults could play a crucial role in improving oral health. In addition, the implementation of preventive measures such as water fluoridation, easier access to toothbrushes and toothpaste, and increased accessibility to dental care services are essential to improve the oral health status of future generations.

## Figures and Tables

**Table 1:** Population characteristics.

	San Cristobal	Valle Escondido	Total	*p* Value
	*n* = 60	*n* = 46	*n* = 106	
	mean (SD)	mean (SD)	mean (SD)	
Age	8.7 (1.7)	8.6 (1.8)	8.7 (1.7)	0.600
BMI	17.8 (2.1)	19 (3.2)	18.3 (2.7)	0.018
	*n* (%)	*n* (%)	*n* (%)	
Systemic diseases ^	2 (3.3)	2 (4.2)	4 (3.8)	0.787
Regular medication use *	0	3 (6.5)	3 (2.8)	0.079
Type of dentition				0.156
Primary dentition	0	2 (4.3)	2 (1.9)	
Mixed dentition	44 (73.3)	30 (65.2)	74 (69.8)	
Permanent dentition	16 (26.7)	14 (30.4)	30 (28.3)	
First dental examination	40 (66.7)	41 (89.1)	81 (76.4)	0.007
Daily toothbrushing	43 (71.7)	26 (56.5)	69 (65.1)	0.105
Sugar intake outside of main meals	59 (98.3)	46 (100)	105 (99.1)	0.284
Daily intake of soda drinks	17 (28.3)	9 (19.6)	26 (24.5)	0.298
Daily intake of hot chocolate	31 (51.7)	10 (21.7)	41 (38.7)	0.002
Daily intake of candies	19 (31.7)	9 (19.6)	28 (26.4)	0.161

SD = standard deviation; ^ = systemic diseases included: chronic nose bleeds, chronic knee pain, and one child was mute; * = which medication is unknown as patients could not remember the names of medications.

**Table 2:** Plaque index in the study population.

	San Cristobal	Valle Escondido	Total
	*n* = 60	*n* = 46	*n* = 106
PI, mean (SD)	2.8 (0.8)	2.9 (0.7)	2.8 (0.8)
Excellent hygiene (PI = 0), *n* (%)	6 (10.0)	2 (4.3)	8 (7.5)
Good hygiene (PI = 0.1–0.9), *n* (%)	9 (15.0)	8 (17.4)	17 (16.0)
Fair hygiene (PI = 1.0–1.9), *n* (%)	35 (58.3)	30 (65.2)	65 (61.3)
Poor hygiene (PI = 2.0–3.0), *n* (%)	10 (16.7)	6 (13.00)	16 (15.1)

PI = plaque index.

**Table 3:** Caries figures in the study population.

	San Cristobal	Valle Escondido	Total	*p* Values
	*n* = 60	*n* = 46	*n* = 106	
	mean (SD)	mean (SD)	mean (SD)	
Prevalence of children with caries, *n* (%)	48 (80.0)	36 (78.3)	84 (79.2)	0.827
DMFT/dmft in children with caries	3.7 (2.5)	4.6 (2.9)	4.05 (2.7)	0.184
DMFT/dmft in the entire sample	3.1 (2.6)	3.6 (3.2)	3.3 (2.9)	0.520
D/d	2.9 (2.6)	3.3 (2.1)	3.1 (2.8)	0.497
M/m	0.1 (0.1	0.2 (0.7)	0.1 (0.5)	0.189
F/f	0.2 (0.7)	0.2 (1.0)	0.2 (0.8)	0.765
* DMFT in the entire sample	0.5 (0.8)	0.8 (1.4)	0.6 (1.1)	0.945
DT	0.5 (0.8)	0.6 (1.2)	0.6 (1.0)	0.672
MT	0.0 (0.1)	0.0 (0.1)	0.0 (0.1)	0.850
FT	0	0.1 (0.4)	0.0 (0.3)	0.105
* dmft in the entire sample	3.5 (2.7)	4.2 (3.2)	3.8 (2.9)	0.378
dt	2.4 (2.7)	2.7 (3.2)	2.51 (2.9)	0.887
mt	0	0.1 (0.7)	0.1 (0.4)	0.105
ft	0.2 (0.7)	0.1 (0.7)	0.2 (0.7)	0.437

SD = standard deviation; *N* (%) = number of children and percentage of total; SD = standard deviation; N/A = not applicable; D/d = decayed; M/m = missing due to caries; F/f = filled. * Note that the mean number of total primary teeth was 8 and the mean number of total permanent teeth was 16 in this study population.

**Table 4:** Orthodontic characteristics in study population.

	San Cristobal	Valle Escondido	Total	*p* Value
	*n* = 60 *n* (%)	*n* = 44 *n* (%)	*n* = 104 *n* (%)	
Sagittal Molar Class				0.122
Class I	47 (78.3)	36 (81.8)	83 (78.3)	
Class II	4 (6.7)	6 (13.0)	10 (9.4)	
Class III	9 (15.0)	2 (4.3)	11 (10.4)	
Anterior open bite	5 (8.3)	6 (13.0)	11 (10.4)	0.521
Lateral crossbite in habitual occlusion				0.230
Absent	56 (93.3%)	44 (97.8)	99 (93.4)	
Unilateral	4 (6.7)	1 (2.2)	5 (4.7)	
Bilateral	0	0	0	
Anterior crossbite	1 (1.7)	2 (4.3)	2 (1.9)	0.578
Scissor bite	0	0	0	

AOB = anterior open bite. One patient had no permanent teeth. For primary teeth, orthodontic outcomes could not be measured

## Data Availability

All data used during this study are included in this published article as an Appendix A.

## Abbreviations

AOB	Anterior open-bite
BASCD	British Association of the Study Community Dentistry
DDE	Dental defects of the enamel
DMFT	Decayed, missing due to caries, and filled teeth (permanent teeth)
dmft	Decayed, missing due to caries, and filled teeth (primary teeth)
FDI	Federation Dental International
MINSA	Ministry of Health in Panama
NGO	Non-governmental organization
SD	Standard deviation
WHO	World Health Organization

## Appendix A

Original Spanish questionnaire and translation in English administered to children to obtain demographic, oral hygiene, and sugar intake data.

1	Cuál es tu nombre y apellido?/ Cual es tu nombre completo? • What is your name and last name?/What is your full name?
2	Cuál es tu fecha de nacimiento?/ Cuando es tu cumpleaños?/ Cuando naciste? • What is your date of birth?/When is your birthday?/When were you born?
3	Te cepillas los dientes por lo menos una vez al día?/ Tu cepillas los dientes todos los días? • Do you brush your teeth at least once a day?/Do you brush your teeth every day?
4	Comes dulces o tomas bebidas con azúcar durante el día? Por ejemplo: confites, galletas, chocolates, caramelos, duros, soda, refresco, pinolio, café con azúcar, chocolito. • Do you eat candy or drink water during the day? For example: candy, cookies, chocolates, caramel, soda, refresco, soda, pinolio, coffee with sugar, chocolito
5	Tomas soda cada dia?/diario?/todos los dias? • Do you drink soda each day?/daily?/everyday?
6	Tomas chocolito cada dia?/ diario?/ todos los dias? • Do you drink Chocolito each day?/daily?/everyday?
7	Tomas confites cada dia?/ diario?/ todos los dias? • Do you eat candy each day?/daily?/everyday?
8	Tienes una problema de salud?/Tienes problemas médicos?/ Has visitado al médico regularmente? • Do you have health problems?/Do you have medical problems?/Have you visited the doctor regularly?
9	Sabes que enfermedad tienes?/ Sabes el nombre de la enfermedad? • Do you know what disease you have?/Do you know the name of the disease?
10	Tomas pastillas diarias?/ Tomas algunas medicamentos regularmente? • Do you take pills daily?/Do you take medication regularly?
11	Sabes el nombre del medicamento?/ Como se llama los pastillas? • Do you know the name of the medication?/What is the name of the pills?
12	Has visitado una dentista antes?/ Es tu primera vez al dentista?/ Has ido al dentista antes? • Have you visited a dentist before?/Is this your first time at a dentist?/ Have you gone to a dentist before?
13	Tu primera visita con la dentista era con los doctores flotantes?/ Fuiste al dentista con doctores flotantes antes? • What your first dental visit with Floating Doctors?/Did you go to a Floating Doctor’s dentist before?
